# Supplementary material for: Engineering Extracellular Vesicle Production Through Magnetic Ion Channel Activation for Bone Regeneration
Source: Adv Healthc Mater. 2026 Mar 9;15(18):e04542. doi: 10.1002/adhm.202504542 (PMC13176524; doi:10.1002/adhm.202504542)
Supplement: Supplementary file 1 — Supporting File: adhm70978‐sup‐0001‐SuppMat.docx. [file ADHM-15-0-s001.docx]

((Supporting Information can be included here using this template))

Copyright WILEY-VCH Verlag GmbH & Co. KGaA, 69469 Weinheim, Germany, 2013.

**Supplementary information**

**
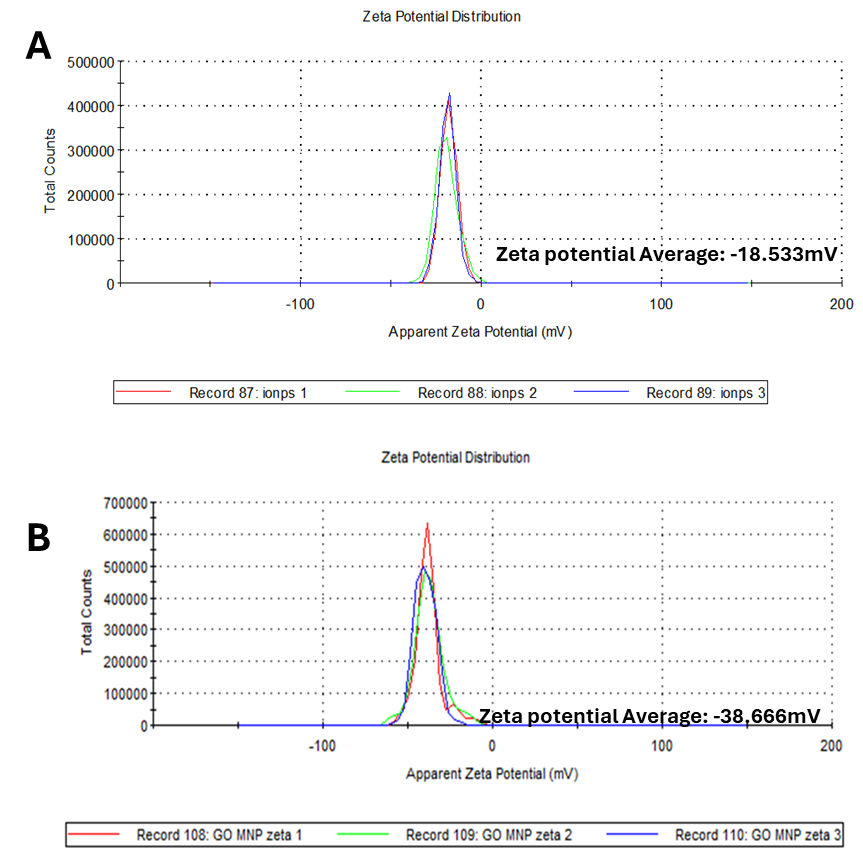
**

**Supplementary Figure 1**. Zeta potential values of A) MNPs and B) GO-MNPs


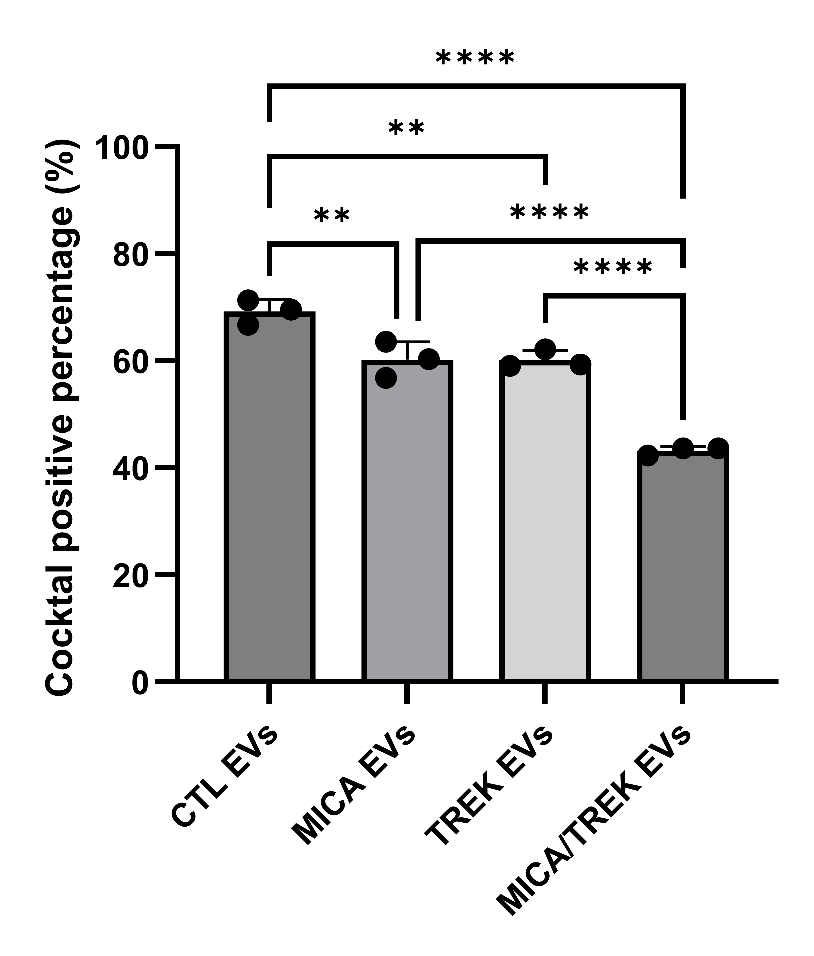


**Supplementary Figure 2**. Cocktail positive percentage of CD9, CD63, and CD81 of isolated EVs. Data are expressed as mean ± SD (n = 3).

**Supplementary Table 1. Differentially enriched proteins within the MICA/TREK EVs.**

| **Protein Name** | **Gene Name** | **Log2fold** | **Anova (p)** |
| --- | --- | --- | --- |
| Nucleotide-binding oligomerization domain-containing protein 2 | NOD2 | 10.04 | 6.21E-11 |
| Fragile X mental retardation protein 1 | FMRP | 3.62 | 1.44E-04 |
| Collagen alpha-3(VI) chain | COL6A3 | 2.94 | 5.45E-06 |
| Centromere-associated protein E | CENPE | 5.03 | 9.91E-07 |
| Histone H4 | H4 | 4.94 | 6.16E-07 |
| MDS1/EVI1 | MECOM | 3.79 | 7.82E-07 |
| Nucleolar protein 4 | NOL4 | 3.58 | 6.31E-07 |
| Tetraspanin-16 | TSPAN16 | 5.49 | 3.08E-07 |
| Malate dehydrogenase_ cytoplasmic | MDH1 | 6.25 | 3.75E-07 |
| Bromodomain-containing protein 9 | BRD9 | 3.07 | 8.75E-06 |
| Annexin A4 | ANXA4 | 2.71 | 9.80E-06 |
| Neurofilament heavy polypeptide | NEFH | 6.14 | 2.27E-04 |
| Fibrillin-1 | FBN1 | 5.70 | 2.55E-07 |
| FERM and PDZ domain-containing protein 3 | FRMD3 | 2.76 | 1.97E-06 |
| Fibronectin | FN1 | 4.88 | 2.91E-07 |
| Zinc finger protein 614 | ZNF614 | 3.87 | 2.02E-02 |
| Keratin_ type I cuticular Ha7 | KRT37 | 3.20 | 2.34E-05 |
| Zinc finger protein 518A | ZNF518A | 4.29 | 2.96E-06 |
| Tubulin beta-2A chain | TUBB2A | 10.13 | 1.81E-07 |
| Protein Daple | CCDC88C | 1.96 | 6.09E-02 |
| Teneurin-1 | TEN1 | 7.30 | 6.36E-07 |
| Ras-related protein Rap-1b | RAP1B | 3.69 | 7.60E-07 |
| POTE ankyrin domain family member E | POTEE | 3.04 | 1.57E-05 |
| 14-3-3 protein theta | YWHAQ | 5.09 | 3.19E-06 |
| Moesin | MSN | 2.52 | 3.40E-06 |
| Annexin A5 | ANXA5 | 2.32 | 5.00E-05 |
| Mitogen-activated protein kinase kinase kinase 13 | MAP4K3 | 4.69 | 4.54E-07 |
| Basement membrane-specific heparan sulfate proteoglycan core protein | HSPG2 | 3.04 | 5.75E-06 |
| Tau-tubulin kinase 1 | TTBK1 | 4.26 | 2.88E-06 |
| Transcription regulator protein BACH2 | BACH2 | 3.03 | 3.90E-05 |
| Semaphorin-3D | SEMA3D | 2.53 | 1.13E-03 |
| Amyloid beta A4 protein | APP | 2.72 | 4.91E-03 |
| Myosin-10 | MYO10 | 2.80 | 9.00E-05 |
| Nuclear receptor corepressor 2 | NCOR2 | 6.59 | 4.39E-07 |
| Ubiquitin-conjugating enzyme E2 H | UBE2H | 3.64 | 2.17E-06 |
| Annexin A6 | ANXA6 | 2.95 | 8.81E-06 |
| Alpha-2-HS-glycoprotein | AHSG | 2.57 | 9.54E-05 |
| Protein Red | IK | 3.02 | 1.99E-05 |
| Proteasome-associated protein ECM29 homolog | ECPAS | 3.46 | 1.22E-04 |
| Nesprin-2 | NESP2 | 3.11 | 3.38E-06 |
| Nephronectin | NPNT | 3.34 | 2.66E-06 |
| Collagen alpha-2(VI) chain | COL6A2 | 7.61 | 4.76E-06 |
| Matrin-3 | MATR3 | 2.06 | 5.56E-04 |
| 60S acidic ribosomal protein P2 | RPLP2 | 3.93 | 7.73E-06 |
| Phospholipid-transporting ATPase | ATP | 7.35 | 4.37E-03 |
| Unconventional prefoldin RPB5 interactor 1 | URI | 9.31 | 1.90E-05 |
| Vinculin | VCL | 6.15 | 1.66E-03 |
| Nuclear autoantigenic sperm protein | NASP | 5.95 | 3.26E-06 |
| Metalloproteinase inhibitor 2 | TIMP2 | 3.47 | 2.49E-06 |
| Nucleosome-remodeling factor subunit BPTF | BPTF | 4.37 | 2.23E-04 |
| Cleavage stimulation factor subunit 3 | CSTF3 | 5.38 | 1.23E-05 |
| Sperm flagellar protein 2 | SPEF2 | 5.87 | 3.53E-03 |
| Guanine nucleotide-binding protein G(s) subunit alpha isoforms XLas | GNAS | 9.92 | 2.83E-07 |
| Putative heat shock protein HSP 90-beta 2 | HSP90B1 | 1.73 | 1.28E-04 |
| 60S ribosomal protein L7 | RPL7 | 3.89 | 4.00E-03 |
| Testis-expressed sequence 2 protein | TEX2 | 8.33 | 9.99E-05 |
| Sema domain_ transmembrane domain (TM)_ and cytoplasmic domain_6A_ isoform CRA_d | SEMA6B | 1.96 | 1.76E-03 |
| Protein disulfide-isomerase A5 | PDIA5 | 5.44 | 5.77E-06 |
| Heat shock protein HSP 90-alpha A2 | HSP90AA2P | 5.91 | 2.96E-05 |
| Metallothionein-1E | MT1E | 3.89 | 1.87E-04 |
| Metallothionein-1X | MT1X | 4.77 | 6.81E-04 |
| Golgi-associated plant pathogenesis-related protein 1 | GLIPR2 | 2.25 | 1.64E-05 |
| Prothymosin alpha | PTMA | 5.75 | 1.32E-05 |
| 60S acidic ribosomal protein P2 | RPLP2 | 3.97 | 5.10E-06 |
| 14-3-3 protein epsilon | YWHAE | 3.05 | 8.18E-03 |
| DNA endonuclease RBBP8 | RBBP8 | 4.29 | 5.52E-04 |
| Elongation factor 1-gamma | EEF1G | 1.83 | 1.14E-05 |
| Periostin | POSTN | 1.93 | 6.35E-04 |
| Nuclear pore complex protein Nup98-Nup96 | NUP98 | 1.09 | 1.05E-03 |
| RAS protein activator like-3 | RASAL3 | 6.41 | 6.10E-05 |
| Beta-arrestin-1 | ARRB1 | 3.68 | 8.63E-06 |
| Myocyte-specific enhancer factor 2B | MEF2B | 4.81 | 8.66E-05 |
| Microtubule-associated protein 2 | MAP2 | 2.80 | 6.06E-05 |
| EGF-like repeat and discoidin I-like domain-containing protein 3 | EDIL3 | 3.77 | 1.35E-06 |
| AF4/FMR2 family member 3 | AFF3 | 4.19 | 9.50E-07 |
| Prelamin-A/C | LMNA | 4.47 | 1.02E-06 |
| Protocadherin | FAT3 | 3.94 | 1.04E-04 |
| Serine/threonine-protein kinase | PLK1 | 8.31 | 6.39E-07 |
| Fermitin family homolog 2 | FERMT2 | 7.35 | 2.33E-05 |
| Centrosomal protein of 192 kDa | CEP192 | 2.13 | 3.20E-04 |
| Ras-related protein Rab-15 | RAB15 | 5.63 | 9.20E-05 |
| E3 ubiquitin-protein ligase | TTC3 | 2.69 | 5.29E-05 |
| Lymphocyte antigen 75 | LY75 | 1.40 | 1.27E-05 |
| MORC family CW-type zinc finger protein 3 | MORC3 | 4.15 | 6.86E-05 |
| 14-3-3 protein eta | YWHAH | 2.63 | 4.56E-03 |
| Putative protein FAM10A4 | ST13P4 | 3.13 | 2.88E-05 |
| Hsc70-interacting protein | ST13 | 3.13 | 2.88E-05 |
| Galactokinase | GALK1 | 4.61 | 8.85E-04 |
| Protein kinase C and casein kinase substrate in neurons protein 3 | PACSIN3 | 10.23 | 8.22E-06 |
| Zinc finger and SCAN domain-containing protein 5A | ZSCAN5A | 11.80 | 2.95E-08 |
| Phosphatidylinositol 4-phosphate 3-kinase C2 domain-containing subunit beta | PIK3C2B | 3.10 | 5.28E-03 |
| Hormone-sensitive lipase | LIPE | 4.06 | 3.27E-06 |
| 60S ribosomal protein L3 | RPL3 | 2.45 | 7.21E-04 |
| Talin-1 | TLN1 | 3.16 | 9.98E-05 |
| UV-stimulated scaffold protein A | UVSSA | 3.02 | 1.44E-04 |
| Alpha-crystallin B chain | CRYAB | 3.06 | 1.66E-06 |
| Cortactin-binding protein 2 | CTTNBP2 | 2.75 | 1.56E-03 |
| Dermcidin | DCD | 2.95 | 2.02E-05 |
| Keratin_ type I cuticular Ha3-II | KRT33B | 3.19 | 6.93E-06 |
| EF-hand calcium-binding domain-containing protein 2 | EFCAB2 | 1.16 | 1.35E-03 |
| 116 kDa U5 small nuclear ribonucleoprotein component | EFTUD2 | 3.99 | 2.54E-07 |
| Zinc finger protein 366 | ZNF366 | 5.51 | 9.02E-05 |
| Keratin_ type II cytoskeletal 74 | KRT74 | 5.95 | 3.12E-04 |
| Zinc finger ZZ-type and EF-hand domain-containing protein 1 | ZZEF1 | 7.73 | 2.04E-05 |
| Testis- and ovary-specific PAZ domain-containing protein 1 | TOPAZ1 | 5.47 | 3.17E-04 |
| Clavesin-2 | CLVS2 | 1.39 | 3.35E-02 |
| High mobility group protein B1 | HMGB1 | 4.21 | 1.03E-04 |
| Heat shock 70 kDa protein 1-like | HSPA1L | 1.90 | 7.92E-04 |
| Solute carrier family 44 member 2 | SLC44A2 | 3.84 | 1.86E-03 |
| Mitogen-activated protein kinase kinase kinase 3 | MAP3K3 | 1.62 | 3.06E-04 |
| Serine/threonine-protein phosphatase PP1-alpha catalytic subunit | PPP1CA | 4.90 | 1.44E-04 |
| Vitamin K-dependent protein S | PROS1 | 2.23 | 1.12E-05 |
| Zinc finger protein 827 | ZNF827 | 6.35 | 1.92E-04 |
| Monocarboxylate transporter 4 | SLC16A3 | 6.38 | 6.81E-04 |
| Male-specific lethal 3 homolog | MSL3 | 5.57 | 3.05E-06 |
| Phosphatidylinositol 3-kinase catalytic subunit type 3 | PIK3C3 | 9.14 | 3.46E-05 |
| Histone H1oo | H1FOO | 2.17 | 9.64E-08 |
| Sodium/potassium-transporting ATPase subunit alpha-1 | ATP1A1 | 11.64 | 1.08E-05 |
| Baculoviral IAP repeat-containing protein 1 | NAIP | 8.03 | 8.58E-05 |
| Guanine nucleotide-binding protein G(t) subunit alpha-1 | GNAT1 | 2.09 | 3.99E-03 |
| G patch domain-containing protein 8 | GPATCH8 | 4.07 | 6.61E-03 |
| Chloride intracellular channel protein 4 | CLIC4 | 2.53 | 2.98E-04 |
| Nascent polypeptide-associated complex subunit alpha | NACA | 4.70 | 2.98E-05 |
| Cyclic nucleotide-gated cation channel beta-3 | CNGB3 | 7.84 | 3.76E-05 |
| Zinc finger protein 521 | ZNF521 | 4.75 | 1.05E-04 |
| Dihydropyrimidinase-related protein 2 | DPYSL2 | 3.91 | 2.85E-05 |
| Cyclin-dependent kinase 13 | CDK13 | 5.30 | 1.37E-04 |
| Coagulation factor X | F10 | 5.18 | 1.66E-05 |
| High mobility group nucleosome-binding domain-containing protein 5 | HMGN5 | 4.14 | 1.22E-04 |
| Leucine zipper putative tumor suppressor 2 | LZTS2 | 6.77 | 1.60E-07 |
| Keratin_ type II cytoskeletal 3 | KRT3 | 4.44 | 9.60E-04 |
| Voltage-dependent T-type calcium channel subunit alpha-1I | CACNA1I | 1.84 | 2.00E-06 |
| Protein shisa-7 | SHISA7 | 16.61 | 4.18E-05 |
| Exocyst complex component 8 | EXOC8 | 3.60 | 4.62E-03 |
| Vacuolar protein sorting-associated protein 18 homolog | VPS18 | 3.81 | 1.95E-03 |
| 60S ribosomal protein L5 | RPL5 | 2.28 | 5.01E-02 |
| Lebercilin-like protein | LCA5L | 2.54 | 6.23E-06 |
| Proliferation-associated protein 2G4 | PA2G4 | 2.22 | 8.61E-05 |
| WD repeat and FYVE domain-containing protein 3 | WDFY3 | 3.04 | 1.09E-03 |
| Ankyrin-2 | ANK2 | 7.24 | 1.30E-03 |
| Nucleoporin NUP188 homolog | NUP188 | 1.42 | 1.09E-04 |
| Ectonucleotide pyrophosphatase/phosphodiesterase family member 2 | ENPP2 | 2.73 | 3.25E-03 |
| HLA class I histocompatibility antigen_ A-80 alpha chain | HLA-A | 5.58 | 1.68E-06 |
| Keratin_ type II cytoskeletal 4 | KRT4 | 4.27 | 7.14E-05 |
| Guanine nucleotide-binding protein G(olf) subunit alpha | GNAL | 3.13 | 4.42E-04 |
| Zinc finger protein 710 | ZNF710 | 4.23 | 6.07E-06 |
| Ubiquitin carboxyl-terminal hydrolase 48 | USP48 | 3.09 | 3.85E-02 |
| Guanine nucleotide-binding protein G(I)/G(S)/G(T) subunit beta-1 | GNB1 | 2.53 | 3.43E-04 |
| Myosin-7B | MYH7B | 2.78 | 1.60E-03 |
| DnaJ homolog subfamily B member 6 | DNAJB6 | 7.24 | 7.55E-05 |
| Fibroblast growth factor receptor 3 | FGFR3 | 6.54 | 5.00E-04 |
| Disintegrin and metalloproteinase domain-containing protein 10 | ADAM10 | 3.78 | 1.12E-03 |
| Histone acetyltransferase KAT6A | KAT6A | 1.24 | 4.65E-04 |
| Desmoglein-1 | DSG1 | 2.12 | 1.21E-07 |
| HLA class I histocompatibility antigen_ A-3 alpha chain | HLA-A | 6.19 | 4.79E-03 |
| BTB/POZ domain-containing protein KCTD17 | KCTD17 | 6.90 | 3.02E-02 |
| Keratin_ type II cytoskeletal 5 | KRT5 | 4.44 | 9.79E-05 |
| Pseudouridylate synthase 7 homolog | PUS7 | 1.67 | 2.12E-05 |
| Heat shock protein HSP 90-alpha | HSP90AA1 | 7.17 | 2.83E-03 |
| Keratin_ type I cuticular Ha1 | KRT31 | 2.78 | 5.00E-06 |
| Protein S100-A11 | S100A11 | 7.64 | 3.15E-04 |
| Deleted in lung and esophageal cancer protein 1 | DLEC1 | 3.94 | 2.59E-06 |
| Keratin_ type II cuticular Hb4 | KRT84 | 6.49 | 1.05E-04 |
| Heterogeneous nuclear ribonucleoprotein H | HNRNPH1 | 5.89 | 4.28E-02 |
| Protein 4.1 | EPB41 | 1.31 | 2.65E-03 |
| Tubulin beta-2B chain | TUBB2B | 7.63 | 1.80E-03 |
| Sodium/potassium-transporting ATPase subunit alpha-2 | ATP1A2 | 3.79 | 3.04E-05 |
| Transforming protein RhoA | RHOA | 4.66 | 6.00E-05 |
| Keratin_ type I cytoskeletal 25 | KRT25 | 1.38 | 1.60E-02 |
| CWF19-like protein 2 | CWF19L2 | 1.08 | 1.03E-03 |
| Calpain-7 | CAPN7 | 6.70 | 4.30E-07 |
| Putative heat shock protein HSP 90-beta-3 | HSP90AB3P | 10.49 | 2.24E-04 |
| MORC family CW-type zinc finger protein 1 | MORC1 | 3.98 | 8.06E-04 |
| Ras-related protein Rab-3B | RAB3B | 1.79 | 1.01E-02 |
| Elongator complex protein 3 | ELP3 | 4.19 | 4.19E-07 |
| Profilin-1 | PFN1 | 4.83 | 1.28E-03 |
| Laminin subunit beta-2 | LAMB2 | 4.53 | 9.04E-05 |
| LIM and senescent cell antigen-like-containing domain protein 1 | LIMS1 | 3.30 | 4.04E-05 |
| Probable aminopeptidase | NPEPL1 | 7.15 | 1.43E-03 |
| Coagulation factor V | F5 | 2.58 | 2.06E-08 |
| Syntaxin-8 | STX8 | 6.28 | 1.38E-04 |
| Keratin_ type II cytoskeletal 6B | KRT6B | 2.81 | 6.70E-06 |
| 60S ribosomal protein L18a | RPL18A | 2.76 | 1.16E-04 |
| Zinc finger FYVE domain-containing protein 26 | ZFYVE26 | 1.27 | 1.74E-04 |
| Lactadherin | MFGE8 | 3.03 | 7.67E-05 |
| DNA excision repair protein | ERCC6 | 2.51 | 1.50E-02 |
| DNA-directed RNA polymerase I subunit RPA34 | CD3EAP | 1.52 | 1.36E-05 |
| Radixin | RDX | 7.25 | 3.45E-04 |
| Galectin-1 | LGALS1 | 4.49 | 3.50E-06 |
| Eukaryotic translation initiation factor 3 subunit A | EIF3A | 4.89 | 6.98E-06 |
| 60S ribosomal protein L4 | RPL4 | 3.12 | 7.52E-05 |
| Ferritin heavy chain | FTH1 | 5.38 | 3.78E-03 |
| Protein bassoon | BSN | 1.07 | 1.72E-04 |
| Ras-related protein Rab-4B | RAB4B | 8.51 | 1.31E-04 |
| Peptidyl-prolyl cis-trans isomerase | PPIE | 1.63 | 2.03E-04 |
| Polyamine-modulated factor 1-binding protein 1 | PMFBP1 | 2.63 | 2.21E-04 |
| Vascular endothelial growth factor receptor 1 | FLT1 | 1.70 | 1.24E-07 |
| Collagen alpha-1(I) chain | COL1A1 | 4.95 | 1.32E-05 |
| STE20-like serine/threonine-protein kinase | SLK | 6.30 | 5.14E-04 |
| Alpha-actinin-4 | ACTN4 | 6.76 | 1.24E-04 |
| Zinc finger and SCAN domain-containing protein 32 | ZSCAN32 | 4.37 | 8.27E-04 |
| Ras-related protein Rab-39A | RAB39A | 4.15 | 2.21E-04 |
| Annexin A2 | ANXA2 | 7.81 | 1.51E-04 |
| Lactotransferrin | LTF | 2.48 | 1.63E-04 |
| Keratin_ type I cytoskeletal 9 | KRT9 | 1.59 | 3.26E-06 |
| Fibrinogen gamma chain | FGG | 2.97 | 3.63E-05 |
| Coiled-coil domain-containing protein 183 | CCDC183 | 3.69 | 2.81E-04 |
| CCAAT/enhancer-binding protein zeta | CEBPZ | 4.29 | 4.48E-04 |
| Serine/threonine-protein kinase PRP4 homolog | PRPF4B | 1.04 | 4.35E-04 |
| T-lymphoma invasion and metastasis-inducing protein 2 | TIAM2 | 1.85 | 3.59E-05 |
| L-lactate dehydrogenase C chain | LDHC | 2.20 | 7.86E-05 |
| Coiled-coil domain-containing protein 157 | CCDC157 | 2.01 | 4.85E-04 |
| Guanine nucleotide-binding protein G(i) subunit alpha-1 | GNAI1 | 3.05 | 3.29E-05 |
| Kalirin | KALRN | 8.00 | 6.30E-03 |
| Zinc finger protein Aiolos | IKZF3 | 1.64 | 1.75E-03 |
| t-SNARE domain containing 1 | TSNARE1 | 3.06 | 2.58E-04 |
| Prothrombin | F2 | 6.45 | 3.03E-03 |
| Nucleotide-binding oligomerization domain-containing protein 1 | NOD1 | 1.37 | 3.16E-04 |
| Fibulin-1 | FBLN1 | 4.63 | 1.07E-04 |
| Pleiotrophin | PTN | 2.30 | 2.63E-03 |
| Ribosomal protein L19 | RPL19 | 3.62 | 8.67E-04 |
| Programmed cell death 6-interacting protein | PDCD6IP | 4.43 | 4.30E-04 |
| Transient receptor potential cation channel subfamily M member 3 | TRPM3 | 2.51 | 2.60E-04 |
| Calcium/calmodulin-dependent protein kinase kinase 1 | CAMKK1 | 6.08 | 1.72E-07 |
| Exportin-6 | XPO6 | 14.45 | 6.73E-02 |
| Stromelysin-1 | MMP3 | 4.43 | 2.50E-03 |
| General transcription factor 3C polypeptide 1 | GTF3C1 | 5.42 | 6.67E-04 |
| Protein S100-A10 | S100A10 | 3.39 | 2.11E-04 |
| 14-3-3 protein sigma | SFN | 6.19 | 1.17E-04 |
| DNA repair and recombination protein RAD54-like | RAD54L | 1.27 | 2.31E-03 |
| Alpha-enolase | ENO1 | 1.13 | 2.29E-03 |
| Keratin_ type II cytoskeletal 1 | KRT1 | 1.35 | 6.45E-05 |
| KIAA0100_ isoform CRA_a | KIAA0100 | 1.20 | 4.46E-06 |
| Mitogen-activated protein kinase kinase kinase | MLT ZAK | 4.48 | 8.96E-03 |
| Serine/threonine-protein phosphatase 6 regulatory subunit 3 | PPP6R3 | 3.87 | 8.03E-02 |
| Prostaglandin E synthase 3 | PTGES3 | 3.25 | 5.43E-04 |
| Alpha-actinin-2 | ACTN2 | 1.50 | 1.39E-02 |
| DNA topoisomerase 2-alpha | TOP2A | 1.90 | 1.08E-03 |
| Guanine nucleotide-binding protein G(i) subunit alpha-2 | GNAI2 | 1.64 | 4.47E-05 |
| Dynein heavy chain 7_ axonemal | DNAH7 | 4.89 | 8.96E-04 |
| RB1-inducible coiled-coil protein 1 | RB1CC1 | 4.37 | 2.43E-04 |
| Nucleosome assembly protein 1-like 4 | NAP1L4 | 5.27 | 1.52E-03 |
| DPH3 homolog | DPH3 | 3.95 | 3.91E-04 |
| OTU domain-containing protein 7A | OTUD7A | 5.62 | 6.62E-06 |
| Transferrin receptor protein 1 | TFRC | 4.55 | 5.63E-07 |
| Msx2-interacting protein | SPEN | 2.04 | 3.47E-05 |
| Lysine-specific demethylase 5C | KDM5C | 4.85 | 2.49E-02 |
| Dedicator of cytokinesis protein 4 | DOCK4 | 1.30 | 1.10E-04 |
| Inter-alpha-trypsin inhibitor heavy chain H2 | ITIH2 | 3.07 | 3.46E-05 |
| Heat shock 70 kDa protein 6 | HSPA6 | 6.44 | 6.24E-05 |
| Keratin_ type II cytoskeletal 80 | KRT80 | 4.51 | 1.47E-03 |
| Coiled-coil domain-containing protein 137 | CCDC137 | 3.93 | 2.96E-05 |
| Rabphilin-3A | RPH3A | 6.02 | 1.52E-03 |
| Nipped-B-like protein | NIPBL | 5.16 | 4.35E-03 |
| Spermatogenesis-associated protein 7 | SPATA7 | 7.33 | 4.33E-04 |
| Peptidyl-prolyl cis-trans isomerase F | PPIF | 1.66 | 4.17E-04 |
| Keratin_ type II cytoskeletal 75 | KRT75 | 1.66 | 9.01E-04 |
| Growth arrest-specific protein 6 | GAS6 | 4.32 | 1.34E-03 |
| Nucleosome assembly protein 1-like 1 | NAP1L1 | 11.58 | 2.89E-05 |
| N-acetyl-beta-glucosaminyl-glycoprotein 4-beta-N-acetylgalactosaminyltransferase 1 | B4GALNT4 | 7.83 | 9.58E-03 |
| RAS guanyl-releasing protein 1 | RASGRP1 | 1.92 | 9.24E-03 |
| Vacuolar protein sorting-associated protein 13A | VPS13A | 3.11 | 1.16E-05 |
| Bromodomain-containing protein 4 | BRD4 | 4.38 | 2.45E-06 |
| Rho GTPase-activating protein 20 | ARHGAP20 | 5.39 | 2.82E-04 |
| U6 snRNA-associated Sm-like protein LSm1 | LSM1 | 1.46 | 9.67E-05 |
| GMP reductase | GMPR2 | 8.69 | 9.97E-03 |
| Myosin light chain 6B | MYL6B | 2.03 | 3.91E-02 |
| Phosphoglycerate mutase 1 | PGAM1 | 2.01 | 9.64E-03 |
| Guanine nucleotide-binding protein G(k) subunit alpha | GNAI3 | 2.91 | 9.47E-02 |
| Mast/stem cell growth factor receptor Kit | KIT | 1.46 | 1.31E-04 |
| Ubiquitin-40S ribosomal protein S27a | RPS27A | 1.73 | 1.73E-03 |
| Ubiquitin-60S ribosomal protein L40 | UbUBA52 | 5.08 | 1.73E-03 |
| Macrophage-stimulating protein receptor | MST1R | 5.08 | 9.92E-03 |
| Tyrosine-protein kinase Fer | FER | 5.26 | 6.00E-07 |
| Hemoglobin subunit gamma-2 | HBG2 | 7.94 | 1.86E-05 |
| AMP deaminase 3 | AMPD3 | 6.75 | 5.09E-04 |
| Bcl-2/adenovirus E1B 19 kDa-interacting protein 2-like protein | BNIPL | 5.34 | 4.91E-06 |
| Zinc finger protein 423 | ZNF423 | 2.34 | 1.72E-06 |
| Huntingtin | HTT | 5.67 | 2.01E-06 |
| Histone-lysine N-methyltransferase | SUV420H1 | 8.04 | 3.41E-04 |
| Zinc finger CCCH domain-containing protein 7A | ZC3H7A | 2.27 | 1.78E-02 |
| Myosin-3 | MYH3 | 1.22 | 5.17E-04 |
| Nucleolin | NCL | 4.48 | 2.78E-04 |
| Sorcin | SRI | 3.35 | 1.19E-04 |
| Growth arrest-specific protein 8 | GAS8 | 4.81 | 3.22E-04 |
| Histone H2A type 1-B/E | HIST1H2AB | 1.83 | 6.83E-06 |
| NEDD4-binding protein 2 | N4BP2 | 1.81 | 1.13E-03 |
| Integrin beta-3 | ITGB3 | 1.29 | 1.46E-03 |
| POTE ankyrin domain family member F | POTEF | 2.40 | 9.58E-02 |
| Mitogen-activated protein kinase kinase kinase 12 | MAP3K12 | 1.24 | 1.20E-04 |
| T-lymphoma invasion and metastasis-inducing protein 1 | TIAM1 | 3.47 | 5.76E-05 |
| Glutamine--tRNA ligase | QARS | 2.47 | 4.13E-02 |
| Unconventional myosin-XIX | MYO19 | 1.67 | 5.20E-05 |
| Putative Ras-related protein Rab-1C | RAB1C | 5.12 | 5.24E-04 |
| Plasminogen | PLG | 1.17 | 2.69E-03 |
| Collagen alpha-1(XII) chain | COL12A1 | 4.27 | 4.89E-06 |
| Secreted phosphoprotein 24 | SPP2 | 3.34 | 1.25E-04 |
| Zinc finger protein 283 | ZNF283 | 1.58 | 1.09E-05 |
| Heterogeneous nuclear ribonucleoprotein U | HNRNPU | 1.61 | 1.23E-04 |
| Rod cGMP-specific 3'_5'-cyclic phosphodiesterase subunit beta | PDE6B | 3.53 | 5.18E-03 |
| Serine protease | HTRA1 | 5.48 | 5.54E-05 |
| Jouberin | AHI1 | 3.57 | 4.65E-03 |
| Heat shock-related 70 kDa protein 2 | HSPA2 | 5.48 | 1.77E-02 |
| Fibrous sheath-interacting protein 1 | FSIP1 | 2.30 | 4.12E-03 |
| Integrin-linked protein kinase | ILK | 3.43 | 1.65E-03 |
| Heat shock cognate 71 kDa protein | HSPA8 | 1.43 | 1.03E-02 |
| Filamin A | FLNA | 2.78 | 4.33E-03 |
| Serine protease 23 | PRSS23 | 1.89 | 1.03E-02 |
| Transformation/transcription domain-associated protein | TRRAP | 1.33 | 1.11E-05 |
| Unconventional myosin-Ic | MYO1C | 3.12 | 1.30E-04 |
| Tumor necrosis factor ligand superfamily member 13 | TNFSF13 | 5.49 | 2.99E-03 |
| Integrin beta-1 | ITGB1 | 2.13 | 3.68E-05 |
| Protein SSX1 | SSX1 | 5.24 | 3.70E-04 |
| Keratin_ type II cytoskeletal 7 | KRT7 | 9.44 | 2.77E-03 |
| Apolipoprotein E | APOE | 1.69 | 1.26E-06 |
| Intraflagellar transport protein 172 homolog | IFT172 | 2.21 | 3.74E-02 |
| Latent-transforming growth factor beta-binding protein 3 | LTBP3 | 1.21 | 2.59E-02 |
| Catenin alpha-1 | CTNNA1 | 4.02 | 2.80E-02 |
| Eukaryotic initiation factor 4A-II | EIF4A2 | 1.18 | 1.98E-05 |
| Serine/threonine-protein kinase 24 | STK24 | 4.97 | 2.35E-02 |
| Myomesin-2 | MYOM2 | 2.97 | 9.62E-02 |
| Protein kinase C alpha type | PRKCA | 2.01 | 3.21E-05 |
| Tropomyosin alpha-4 chain | TPM4 | 5.43 | 8.70E-05 |
| Collagen alpha-1(VI) chain | COL6A1 | 3.96 | 9.95E-06 |
| Cell division control protein 42 homolog | CDC42 | 3.51 | 1.58E-03 |
| E3 ubiquitin-protein ligase | TRIM37 | 1.80 | 1.66E-03 |
| Dynein light chain 1_ cytoplasmic | DYNLL1 | 1.22 | 6.73E-08 |
| Keratin_ type II cytoskeletal 72 | KRT72 | 11.90 | 4.76E-03 |
| Dedicator of cytokinesis protein 9 | DOCK9 | 3.31 | 6.58E-03 |
| Fermitin family homolog 3 | FERMT3 | 3.61 | 1.16E-03 |
| 72 kDa type IV collagenase | MMP2 | 2.06 | 1.29E-04 |
| Tyrosine-protein kinase receptor UFO | AXL | 6.73 | 5.40E-03 |
| Kinesin-like protein KIF16B | KIF16B | 3.13 | 3.10E-04 |
| Ephrin type-A receptor 4 | EPHA4 | 2.82 | 1.20E-03 |
| Intron-binding protein aquarius | AQR | 2.20 | 5.60E-04 |
| Pro-neuregulin-1_ membrane-bound isoform | NRG1 | 4.69 | 9.71E-05 |
| Glyceraldehyde-3-phosphate dehydrogenase | GAPDH | 1.67 | 2.38E-02 |
| 14-3-3 protein gamma | YWHAG | 2.42 | 2.66E-03 |
| CAP-Gly domain-containing linker protein 3 | CLIP3 | 1.79 | 1.63E-05 |
| Myosin-14 | MYH14 | 7.85 | 4.72E-06 |
| 40S ribosomal protein S3a | RPS3A | 3.62 | 4.23E-03 |
| FYVE and coiled-coil domain-containing protein 1 | FYCO1 | 1.06 | 1.25E-02 |
| DENN domain-containing protein 4B | DENND4B | 1.34 | 2.25E-03 |
| Chloride intracellular channel protein 1 | CLIC1 | 4.77 | 1.72E-02 |
| Dystrophin | DMD | 4.92 | 7.40E-03 |
| C-type lectin domain family 1 member B | CLEC1B | 3.69 | 8.63E-07 |
| Syntenin-1 | SDCBP | 5.77 | 5.94E-05 |
| Coiled-coil domain-containing protein 144B | CCDC144B | 2.60 | 3.45E-02 |
| Hemoglobin subunit alpha | HBA1 | 2.98 | 3.43E-04 |
| LIM/homeobox protein Lhx2 | LHX2 | 1.31 | 8.79E-05 |
| A-kinase anchor protein 9 | AKAP9 | 1.99 | 1.72E-05 |
| Elongation factor 1-alpha 1 | EEF1A1 | 4.15 | 3.70E-04 |
| Beta-2-glycoprotein 1 | APOH | 2.19 | 7.17E-03 |
| BICD1 protein | BICD1 | 5.10 | 3.93E-06 |
| Ribosomal protein S6 kinase | RPS6KA1 | 4.68 | 1.59E-04 |
| Laminin subunit gamma-1 | LAMC1 | 1.82 | 7.34E-04 |
| Immunoglobulin lambda-like polypeptide 1 | IGLL1 | 6.77 | 4.50E-06 |
| Metallothionein-1G | MT1G | 8.90 | 6.89E-03 |
| Ribosomal protein S6 kinase alpha-3 | RPS6KA3 | 15.60 | 2.49E-04 |
| High affinity cAMP-specific and IBMX-insensitive 3'_5'-cyclic phosphodiesterase 8B | PDE8B | 6.11 | 2.85E-04 |
| Laminin subunit alpha-2 | LAMA2 | 1.92 | 2.92E-04 |
| Coiled-coil domain-containing protein 144A | CCDC144A | 2.75 | 2.21E-02 |
| Dedicator of cytokinesis protein 1 | DOCK1 | 5.63 | 8.75E-03 |
| AP-3 complex subunit beta-1 | AP3B1 | 5.10 | 1.43E-06 |
| Heat shock protein 75 kDa_ mitochondrial | TRAP1 | 14.02 | 4.59E-02 |
| Keratin_ type II cytoskeletal 2 epidermal | KRT2 | 1.97 | 1.15E-06 |
| Heterogeneous nuclear ribonucleoprotein H2 | HNRNPH2 | 4.29 | 3.65E-02 |
| Tropomyosin alpha-3 chain | TPM3 | 1.80 | 3.29E-05 |
| Hemoglobin subunit delta | HBD | 6.67 | 1.50E-05 |
| Keratin_ type I cuticular Ha5 | KRT35 | 4.85 | 2.55E-04 |
| Heat Shock Protein 90 Beta Family Member 1 | HSP90B1 | 1.89 | 5.56E-05 |
| Echinoderm microtubule-associated protein-like 5 | EML5 | 1.64 | 3.16E-03 |
| 60S ribosomal protein L30 | RPL30 | 6.93 | 1.27E-06 |
| Myosin-7 | MYH7 | 7.40 | 1.12E-03 |
| Ras-related protein Rab-3C | RAB3C | 5.19 | 1.15E-03 |
| Laminin subunit alpha-4 | LAMA4 | 3.89 | 9.86E-05 |
| Tubulin alpha-4A chain | TUBA4A | 2.01 | 4.58E-06 |
| Centriolin | CNTRL | 5.97 | 4.81E-04 |
| Keratin_ type I cuticular Ha6 | KRT36 | 4.81 | 6.31E-04 |
| Hemoglobin subunit beta | HBB | 1.94 | 8.72E-05 |
| Fibrinogen beta chain | FGB | 5.05 | 3.71E-04 |
| Nuclear ubiquitous casein and cyclin-dependent kinase substrate 1 | NUCKS1 | 4.78 | 6.73E-04 |
| Prolargin | PRELP | 8.32 | 9.13E-03 |
| Centrosomal protein of 57 kDa | CEP57 | 3.76 | 2.22E-04 |
| MADS box transcription enhancer factor 2_ polypeptide C_ isoform CRA_e | MEF2C | 4.92 | 6.81E-05 |
| Guanine nucleotide-binding protein subunit alpha-11 | GNA11 | 11.00 | 8.31E-07 |
| Myosin-6 | MYH6 | 5.25 | 1.57E-03 |
| Gamma-aminobutyric acid type B receptor subunit 1 | GABBR1 | 3.62 | 5.02E-05 |
| Tyrosine-protein phosphatase non-receptor type 12 | PTPN12 | 10.65 | 4.86E-03 |
| DEP domain-containing protein 5 | DEPDC5 | 4.45 | 3.67E-05 |
| Vitamin D-binding protein | GC | 4.17 | 1.61E-04 |
| Myosin-1 | MYH1 | 5.91 | 1.76E-02 |
| Keratin_ type I cytoskeletal 20 | KRT20 | 3.19 | 1.86E-02 |
| Vascular endothelial growth factor receptor 3 | FLT4 | 3.88 | 2.36E-06 |
| HLA class I histocompatibility antigen_ alpha chain G | HLA-G | 3.68 | 2.41E-02 |
| Unconventional myosin-IXb | MYO9B | 1.27 | 2.26E-02 |
| Dynein heavy chain 1_ axonemal | DNAH1 | 4.81 | 8.11E-05 |
| Filamin-B | FLNB | 4.36 | 9.03E-05 |
| Pre-rRNA-processing protein TSR1 homolog | TSR1 | 6.31 | 1.04E-03 |
| Histone H2B type 1-K | HIST1H2BK | 4.98 | 3.89E-03 |
| Bromodomain adjacent to zinc finger domain protein 2B | BAZ2B | 2.79 | 6.98E-02 |
| Ezrin | EZR | 1.80 | 1.65E-02 |
| Acidic leucine-rich nuclear phosphoprotein 32 family member E | ANP32E | 4.15 | 6.90E-06 |
| Myosin-8 | MYH8 | 7.03 | 3.10E-03 |
| TATA box-binding protein-associated factor RNA polymerase I subunit B | TAF1B | 2.28 | 4.53E-02 |
| 40S ribosomal protein S8 | RPS8 | 2.30 | 2.96E-03 |
| AF4/FMR2 family member 4 | AFF4 | 1.37 | 5.23E-04 |
| EF-hand calcium-binding domain-containing protein 5 | EFCAB5 | 8.78 | 7.35E-02 |
| Ras-related protein Rap-1A | RAP1A | 2.23 | 7.75E-03 |
| Pyruvate kinase PKM | PKM | 1.14 | 2.92E-06 |
| Putative beta-actin-like protein 3 | POTEKP | 2.68 | 6.25E-03 |
| Guanine nucleotide-binding protein G(I)/G(S)/G(T) subunit beta-2 | GNB2 | 3.37 | 6.70E-04 |
| Centrosome and spindle pole-associated protein 1 | CSPP1 | 3.21 | 2.44E-05 |
| Transcription factor 20 | TCF20 | 6.55 | 7.17E-06 |
| Collagen type IV alpha-3-binding protein | COL4A3BP | 4.44 | 6.45E-04 |
| Alpha-actinin-1 | ACTN1 | 4.55 | 3.11E-03 |
| Ninein-like protein | NINL | 2.36 | 2.03E-04 |
| Calmodulin | CALM1 | 3.86 | 8.28E-04 |
| Protein S100-A4 | S100A4 | 3.95 | 1.81E-06 |
| Kelch-like protein 1 | KLHL1 | 4.23 | 1.40E-02 |
| Lysine-specific histone demethylase 1A | KDM1A | 6.04 | 2.57E-04 |
| Tripartite motif-containing protein 26 | TRIM26 | 2.80 | 1.22E-04 |
| L-lactate dehydrogenase A-like 6A | LDHAL6A | 2.63 | 1.25E-02 |
| Transketolase | TKT | 1.97 | 2.22E-02 |
| Probable E3 ubiquitin-protein ligase | DTX2 | 2.01 | 1.64E-02 |
| Nucleoside diphosphate kinase B | NME2 | 2.81 | 1.66E-03 |
| DNA replication licensing factor | MCM4 | 2.38 | 1.08E-07 |
| Period circadian protein homolog 2 | PER2 | 11.93 | 2.78E-05 |
| T-complex protein 1 subunit theta | CCT8 | 6.30 | 4.42E-04 |
| Tektin-1 | TEKT1 | 7.02 | 8.39E-02 |
| Ankyrin and armadillo repeat-containing protein | ANKAR | 2.07 | 5.38E-03 |
| 14-3-3 protein beta/alpha | YWHAB | 3.95 | 2.28E-04 |
| AT-rich interactive domain-containing protein 4A | ARID4A | 2.60 | 2.95E-04 |
| Apolipoprotein B-100 | APOB | 1.48 | 9.29E-06 |
| Synaptic vesicle membrane protein VAT-1 homolog | VAT1 | 2.75 | 6.29E-03 |
| Keratin_ type I cytoskeletal 16 | KRT16 | 3.47 | 9.04E-06 |
| Annexin A11 | ANXA11 | 3.78 | 1.85E-03 |
| Transmembrane protein 98 | TMEM98 | 3.17 | 2.09E-06 |
| Tubulin alpha-1C chain | TUBA1C | 8.12 | 4.34E-05 |
| Sister chromatid cohesion protein PDS5 homolog A | PDS5A | 7.17 | 2.54E-04 |
| Vacuolar protein sorting-associated protein 13C | VPS13C | 7.07 | 7.23E-03 |
| Myosin-4 | MYH4 | 2.91 | 1.02E-02 |
| Rho GTPase-activating protein 18 | ARHGAP18 | 2.63 | 3.16E-06 |
| Actin_ cytoplasmic 1 | ACTB | 1.19 | 6.23E-06 |
| Zinc finger protein 687 | ZNF687 | 2.76 | 9.67E-03 |
| Ribosomal protein L15 | RPL15 | 3.03 | 4.60E-03 |
| Adenosylhomocysteinase | AHCY | 1.90 | 2.28E-04 |
| Reticulon-4 | RTN4 | 3.38 | 5.59E-04 |
| Teneurin-3 | TENM3 | 8.74 | 2.14E-02 |
| 40S ribosomal protein S9 | RPS9 | 2.38 | 3.44E-02 |
| Ras-related protein Rab-5B | RAB5B | 2.07 | 2.23E-02 |
| Ubiquitin carboxyl-terminal hydrolase | USP36 | 1.58 | 5.29E-05 |
| Tubulin polyglutamylase | TTLL5 | 4.25 | 1.49E-03 |
| Ras-related protein Rab-11B | RAB11B | 3.49 | 2.10E-04 |
| Long-chain-fatty-acid--CoA ligase | ACSBG2 | 1.86 | 3.50E-03 |
| Band 4.1-like protein 2 | EPB41L2 | 5.24 | 3.74E-05 |
| Terminal uridylyltransferase 4 | ZCCHC11 | 4.87 | 3.35E-04 |
| Pleckstrin homology domain-containing family J member 1 | PLEKHJ1 | 1.31 | 2.78E-03 |
| mRNA-capping enzyme | RNGTT | 6.56 | 5.83E-02 |
| Guanine nucleotide-binding protein subunit beta-2-like 1 | GNB2L1 | 3.70 | 6.19E-03 |
| Rabenosyn-5 | RBSN | 6.11 | 1.05E-07 |
| Receptor protein-tyrosine kinase | TYRO3 | 9.76 | 1.49E-03 |
| Ubiquitin-conjugating enzyme E2 D3 | UBE2D3 | 1.29 | 3.05E-03 |
| UPF0505 protein | C16orf62 | 1.78 | 2.27E-03 |
| Signal transducer and activator of transcription 6 | STAT6 | 4.85 | 1.40E-04 |
| Rho-related GTP-binding protein RhoB | RHOB | 2.52 | 2.37E-03 |
| Unconventional myosin-If | MYO1F | 3.49 | 1.33E-02 |
| Ras-related protein Rab-7a | RAB7A | 1.89 | 2.59E-05 |
| Ras-related protein Rab-10 | RAB10 | 2.19 | 1.04E-06 |
| Inosine-5'-monophosphate dehydrogenase 1 | IMPDH1 | 5.88 | 6.77E-02 |
| Probable ATP-dependent RNA helicase | DDX20 | 2.33 | 6.69E-04 |
| HLA class I histocompatibility antigen_ Cw-2 alpha chain | HLA-C | 7.49 | 9.27E-06 |
| Histone H3.1t | HIST3H3 | 6.27 | 1.56E-04 |
| Plexin-D1 | PLXND1 | 1.22 | 5.88E-02 |
| Zinc finger protein 16 | ZNF16 | 4.52 | 5.28E-04 |
| Acetyl-CoA carboxylase 1 | ACACA | 4.67 | 1.13E-04 |
| Ribosomal protein S6 kinase beta-2 | RPS6KB2 | 2.63 | 4.51E-06 |
| Mediator of RNA polymerase II transcription subunit 24 | MED24 | 2.97 | 3.89E-05 |
| Katanin p60 ATPase-containing subunit A-like 2 | KATNAL2 | 3.35 | 5.77E-02 |
| HCG1745306_ isoform CRA_a | HBA2 | 1.78 | 2.04E-05 |
| TBC1 domain family member 1 | TBC1D1 | 2.31 | 2.31E-05 |
| Alpha-2-macroglobulin | A2M | 3.31 | 1.65E-04 |
| Cofilin-2 | CFL2 | 1.35 | 1.83E-02 |
| E3 ubiquitin-protein ligase UBR5 | UBR5 | 3.07 | 6.56E-02 |
| Putative heat shock protein HSP 90-alpha A5 | HSP90AA5P | 2.00 | 4.66E-05 |
| Ras-related protein Rab-30 | RAB30 | 3.43 | 2.28E-02 |
| Rab GDP dissociation inhibitor beta | GDI2 | 1.19 | 8.44E-02 |
| Attractin-like protein 1 | ATRNL1 | 1.98 | 3.06E-04 |
| Coiled-coil domain-containing protein 146 | CCDC146 | 3.50 | 4.90E-06 |
| Coagulation factor IX | F9 | 3.82 | 1.26E-05 |
| Coagulation factor XIII A chain | F13A1 | 3.63 | 4.52E-06 |
| E3 ubiquitin-protein ligase CBL | CBL | 8.42 | 7.72E-02 |
| Guanine nucleotide-binding protein subunit alpha-12 | GNA12 | 2.56 | 1.97E-02 |
| Semaphorin-4A | SEMA4A | 1.83 | 2.19E-06 |
| WD repeat-containing protein 60 | WDR60 | 8.42 | 1.47E-05 |
| Claspin | CLSPN | 5.46 | 1.62E-02 |
| Brefeldin A-inhibited guanine nucleotide-exchange protein 2 | ARFGEF2 | 5.15 | 5.96E-05 |
| Keratin_ type I cytoskeletal 18 | KRT18 | 1.06 | 1.95E-05 |
| Ubiquitin-like modifier-activating enzyme | ATG7 | 2.66 | 3.04E-05 |
| L-lactate dehydrogenase B chain | LDHB | 4.50 | 3.69E-03 |
| Acidic leucine-rich nuclear phosphoprotein 32 family member A | ANP32A | 1.66 | 4.45E-02 |
| GTP-binding nuclear protein Ran | RAN | 2.88 | 8.43E-03 |
| Triosephosphate isomerase | TPI1 | 2.28 | 5.37E-06 |
| Dynein heavy chain 14_ axonemal | DNAH14 | 3.85 | 1.10E-02 |
| Ribonuclease H1 | RNASEH1 | 1.58 | 1.03E-07 |
| 60S acidic ribosomal protein P0 | RPLP0 | 6.78 | 6.34E-04 |
| Elongation factor 2 | EEF2 | 2.70 | 8.34E-02 |
| Kinesin-like protein KIF28P | KIF28P | -11.2987 | 2.31E-08 |
| Dihydropyrimidinase-related protein 1 | CRMP1 | -2.37685 | 5.21E-06 |
| Epididymis luminal protein 189 | DKFZp686J1372 | -1.24396 | 1.24E-05 |
| Wings apart-like protein homolog | WAPAL | -4.96751 | 2.36E-07 |
| Gamma-aminobutyric acid receptor subunit rho-1 | GABRR1 | -2.23479 | 4.17E-06 |
| NFX1-type zinc finger-containing protein 1 | ZNFX1 | -4.92847 | 2.60E-06 |
| H2.0-like homeobox protein | HLX | -2.05725 | 5.02E-06 |
| NADPH:adrenodoxin oxidoreductase_ mitochondrial | FDXR | -7.2734 | 2.01E-07 |
| Zinc finger protein 40 | HIVEP1 | -5.34564 | 0.000668 |
| Tyrosine-protein kinase CSK | CSK | -3.04899 | 7.65E-06 |
| DNA topoisomerase I_ mitochondrial | TOP1MT | -13.8424 | 1.22E-06 |
| Zinc finger protein 626 | ZNF626 | -7.94775 | 5.70E-07 |
| RING finger protein 207 | RNF207 | -11.2952 | 8.02E-06 |
| Kinectin | KTN1 | -3.91703 | 9.12E-08 |
| DNA-dependent protein kinase catalytic subunit | PRKDC | -4.40111 | 1.60E-05 |
| Receptor protein-tyrosine kinase | FGFR4 | -13.8654 | 6.37E-06 |
| Bromodomain testis-specific protein | BRDT | -1.22532 | 0.000405 |
| Ankyrin repeat domain-containing protein 6 | ANKRD6 | -3.6318 | 0.001243 |
| Zinc finger protein 671 | ZNF671 | -3.56031 | 0.000204 |
| Keratin_ type II cytoskeletal 78 | KRT78 | -3.14652 | 1.03E-05 |
| Cytoplasmic dynein 2 light intermediate chain 1 | DYNC2LI1 | -13.4216 | 5.29E-06 |
| Cytoplasmic phosphatidylinositol transfer protein 1 | PITPNC1 | -5.43888 | 3.99E-05 |
| DNA replication licensing factor MCM7 | MCM7 | -4.80057 | 2.90E-05 |
| Histidine triad nucleotide-binding protein 1 | HINT1 | -1.37455 | 0.009339 |
| Bridging integrator 3 | BIN3 | -8.48248 | 0.000317 |
| Vitronectin | VTN | -2.86258 | 4.56E-05 |
| Major vault protein | MVP | -2.08711 | 2.73E-05 |
| Heat shock 70 kDa protein 1B | HSPA1B | -3.69879 | 1.25E-06 |
| Keratin_ type II cytoskeletal 1b | KRT77 | -3.10162 | 0.003618 |
| Synaptotagmin-1 | SYT1 | -7.46074 | 1.53E-06 |
| Transforming growth factor-beta receptor-associated protein 1 | TGFBRAP1 | -1.58673 | 2.31E-05 |
| E3 ubiquitin-protein ligase BRE1A | RNF20 | -13.9309 | 0.000103 |
| ADP-ribosylation factor 5 | ARF5 | -2.53397 | 0.003917 |
| Erythrocyte band 7 integral membrane protein | STOM | -3.37638 | 0.000177 |
| Fibulin-1 | FBLN1 | -10.4008 | 0.000231 |
| Obscurin | OBSCN | -7.47334 | 6.76E-07 |
| Deleted in malignant brain tumors 1 protein | DMBT1 | -3.00563 | 5.24E-06 |
| Putative heat shock protein HSP 90-alpha A4 | HSP90AA4P | -1.0604 | 0.000472 |
| Thrombospondin-1 | THBS1 | -2.15287 | 0.000151 |
| Probable G-protein-coupled receptor 179 | GPR179 | -16.8088 | 2.51E-07 |
| Golgin subfamily A member 4 | GOLGA4 | -2.09717 | 9.78E-05 |
| Inactive carboxypeptidase-like protein X2 | CPXM2 | -3.80255 | 3.99E-05 |
| Serine/threonine-protein kinase Nek3 | NEK3 | -6.72427 | 6.26E-05 |
| Alpha-internexin | INA | -1.58251 | 0.000936 |
| ATP synthase subunit beta_ mitochondrial | ATP5B | -3.27813 | 0.030472 |
| Four and a half LIM domains protein 2 | FHL2 | -9.54326 | 4.45E-07 |
| Myosin-11 | MYH11 | -3.96298 | 1.19E-06 |
| Prohibitin | PHB | -2.54788 | 0.003068 |
| Guanine nucleotide-binding protein subunit beta-4 | GNB4 | -1.85066 | 4.89E-05 |
| Neurexin-1-beta | NRXN1 | -3.44744 | 9.88E-06 |
| Minor histocompatibility antigen H13 | HM13 | -1.3988 | 0.001545 |
| Complement component C9 | C9 | -1.16842 | 0.002187 |
| LINE-1 type transposase domain-containing protein 1 | L1TD1 | -1.05498 | 0.013525 |
| Caveolin | CAV1 | -2.02597 | 0.060923 |
| Histone-lysine N-methyltransferase SETD1B | SETD1B | -1.33365 | 0.000143 |
| Angiopoietin-related protein 2 | ANGPTL2 | -4.25013 | 0.000148 |
| HLA class I histocompatibility antigen_ Cw-6 alpha chain | HLA-C | -3.5302 | 0.00077 |
| Ras GTPase-activating-like protein IQGAP2 | IQGAP2 | -1.48829 | 9.41E-05 |
| E3 ubiquitin-protein ligase HUWE1 | HUWE1 | -2.07311 | 0.000134 |
| Solute carrier family 12 member 5 | SLC12A5 | -8.26886 | 0.0004 |
| Guanine nucleotide-binding protein G(z) subunit alpha | GNAZ | -2.71028 | 0.000685 |
| Lysine--tRNA ligase | KARS | -3.51893 | 2.00E-05 |
| Glutamate decarboxylase 1 | GAD1 | -2.61975 | 0.014592 |
| Procollagen C-endopeptidase enhancer 1 | PCOLCE | -2.47267 | 0.002743 |
| Ras-related protein Rab-1A | RAB1A | -1.02024 | 0.00133 |
| Mucolipin 2_ isoform CRA_a | MCOLN2 | -3.02693 | 0.003282 |
| Dedicator of cytokinesis protein 11 | DOCK11 | -4.48341 | 0.001357 |
| Alpha-fetoprotein | AFP | -1.0593 | 0.001168 |
| ADP-ribosylation factor 3 | ARF3 | -1.25338 | 0.016323 |
| Signal-induced proliferation-associated 1-like protein 1 | SIPA1L1 | -4.26698 | 0.00404 |
| Protein NDNF | NDNF | -3.5861 | 0.056094 |
| ADP-ribosylation factor 6 | ARF6 | -1.72927 | 0.006607 |
| Nucleolin | NCL | -3.87146 | 0.001743 |
| Troponin T_ slow skeletal muscle | TNNT1 | -3.28594 | 6.52E-05 |
| Ras GTPase-activating-like protein IQGAP1 | IQGAP1 | -4.98883 | 3.11E-06 |
| Collagen alpha-2(I) chain | COL1A2 | -4.21187 | 1.04E-05 |
| Apolipoprotein A-I | APOA1 | -1.29911 | 0.001044 |
| Zinc finger CCHC domain-containing protein 8 | ZCCHC8 | -2.99466 | 0.032464 |
| Keratin_ type II cytoskeletal 73 | KRT73 | -3.42258 | 0.006581 |
| Periplakin | PPL | -1.3092 | 0.001671 |
| Peptidyl-prolyl cis-trans isomerase A | PPIA | -2.65242 | 3.49E-05 |
| Casein kinase I isoform gamma-3 | CSNK1G3 | -3.4264 | 0.000363 |
| Semaphorin-3D | SEMA3D | -3.9987 | 0.000728 |
| Histone-lysine N-methyltransferase ASH1L | ASH1L | -4.74311 | 4.62E-05 |
| Transient receptor potential cation channel subfamily M member 6 | TRPM6 | -3.05741 | 1.47E-06 |
| Zinc finger protein GLI4 | GLI4 | -7.7467 | 0.00389 |
| Adipocyte enhancer-binding protein 1 | AEBP1 | -2.45873 | 0.000524 |
| E3 ubiquitin-protein ligase NEDD4 | NEDD4 | -11.7909 | 4.84E-05 |
| Tetranectin | CLEC3B | -3.08162 | 0.00044 |
| Endogenous retrovirus group K member 10 Pol protein | ERVK-10 | -2.73701 | 0.056818 |
| Disks large-associated protein 3 | DLGAP3 | -3.8619 | 0.009872 |
| Junctional protein associated with coronary artery disease | KIAA1462 | -4.06526 | 4.34E-06 |
| FACT complex subunit SSRP1 | SSRP1 | -7.64625 | 4.95E-07 |
| Probable E3 ubiquitin-protein ligase MARCH10 | Mar/10 | -5.09228 | 4.49E-05 |
| Retinoblastoma-like protein 1 | RBL1 | -1.96557 | 0.00026 |
| Retrotransposon-like protein 1 | RTL1 | -2.75611 | 0.034298 |
| Heterogeneous nuclear ribonucleoprotein C-like 2 | HNRNPCL2 | -7.05187 | 1.51E-06 |
| Guanine nucleotide-binding protein G(I)/G(S)/G(T) subunit beta-3 | GNB3 | -2.82008 | 0.006753 |
| Teneurin-4 | TENM4 | -3.30487 | 0.00487 |
| Mismatch repair endonuclease PMS2 | PMS2 | -10.024 | 1.15E-05 |
| Matrix-remodeling-associated protein 5 | MXRA5 | -3.94513 | 7.92E-06 |
| Latent-transforming growth factor beta-binding protein 3 | LTBP3 | -2.82119 | 0.000273 |
| Ras-related protein Rab-14 | RAB14 | -6.3603 | 0.000134 |
| Pigment epithelium-derived factor | SERPINF1 | -3.87614 | 0.002238 |
| Serotransferrin | TF | -2.1585 | 0.002069 |
| Ras-related protein Rab-5C | RAB5C | -2.93361 | 0.002162 |
| Annexin | ANXA3 | -4.23808 | 0.00413 |
| Serine/arginine repetitive matrix protein 2 | SRRM2 | -7.42019 | 0.001257 |
| Serine/arginine-rich-splicing factor 7 | SRSF7 | -5.39971 | 4.87E-05 |
| Beta-defensin 112 | DEFB112 | -4.45576 | 0.000135 |
| MAGUK p55 subfamily member 4 | MPP4 | -3.62936 | 0.005681 |
| Latrophilin-1 | LPHN1 | -3.88671 | 7.41E-05 |
| Exocyst complex component 2 | EXOC2 | -4.78587 | 0.017349 |
| Hemoglobin subunit zeta | HBZ | -4.25156 | 0.000513 |
| Trafficking kinesin-binding protein 1 | TRAK1 | -1.69456 | 0.001442 |
| Keratin_ type I cytoskeletal 17 | KRT17 | -2.17874 | 0.000122 |
| Hydroxyacylglutathione hydrolase_ mitochondrial | HAGH | -6.35235 | 1.94E-05 |
| Protocadherin gamma-C4 | PCDHGC4 | -8.53722 | 9.52E-07 |
| Putative Polycomb group protein ASXL3 | ASXL3 | -1.67402 | 0.007715 |
| Tubulin polyglutamylase TTLL7 | TTLL7 | -3.24259 | 0.004981 |
| Cyclin-L1 | CCNL1 | -7.83696 | 2.58E-05 |
| Tubulin beta-1 chain | TUBB1 | -4.04964 | 1.08E-05 |
| Zinc finger protein 638 | ZNF638 | -11.2987 | 0.032204 |
| T-complex protein 1 subunit eta | CCT7 | -2.26364 | 0.000603 |
| T-complex protein 1 subunit beta | CCT2 | -6.38681 | 0.011023 |
| Gamma-aminobutyric acid receptor subunit gamma-3 | GABRG3 | -1.67603 | 0.057783 |
| Transient receptor potential cation channel subfamily M member 2 | TRPM2 | -3.15304 | 0.003987 |
| Zinc finger protein 320 | ZNF320 | -10.2461 | 0.000117 |
| Dual specificity mitogen-activated protein kinase kinase 3 | MAP2K3 | -13.0719 | 1.79E-07 |
| Peroxiredoxin-1 | PRDX1 | -4.8246 | 9.44E-07 |
| Putative tRNA pseudouridine synthase Pus10 | PUS10 | -1.82421 | 0.050015 |
| Brefeldin A-inhibited guanine nucleotide-exchange protein 1 | ARFGEF1 | -8.57428 | 6.08E-06 |
| Pleckstrin homology-like domain family B member 2 | PHLDB2 | -4.76837 | 6.25E-06 |
| Serine/threonine-protein kinase 25 | STK25 | -6.7863 | 0.002037 |
| Isovaleryl-CoA dehydrogenase_ mitochondrial | IVD | -7.61084 | 3.66E-05 |
| Amine oxidase [flavin-containing] A | MAOA | -5.72259 | 0.022826 |
| Transmembrane protein 131-like | KIAA0922 | -2.10775 | 0.057208 |
| Zinc finger protein 432 | ZNF432 | -6.40177 | 0.005427 |
| AF4/FMR2 family member 2 | AFF2 | -1.40982 | 0.089088 |
| Glyoxalase domain-containing protein 5 | GLOD5 | -2.73731 | 0.000757 |
| Glutamyl aminopeptidase | ENPEP | -3.87776 | 0.015569 |
| Reticulocalbin-3 | RCN3 | -3.86025 | 0.014171 |
| Ras-related protein Rab-39B | RAB39B | -3.89586 | 0.000833 |
| Terminal uridylyltransferase 7 | ZCCHC6 | -2.53704 | 0.036366 |
| Transcription factor TFIIIB component B'' homolog | BDP1 | -2.32497 | 2.05E-05 |
| Vesicle-fusing ATPase | NSF | -3.43592 | 0.019775 |
| Apolipoprotein M | APOM | -1.66837 | 0.06297 |
| Vimentin | VIM | -1.71933 | 0.050469 |
| Transitional endoplasmic reticulum ATPase | VCP | -5.04267 | 0.065533 |
| Collagen alpha-1(III) chain | COL3A1 | -3.34153 | 0.009644 |
| Neurofibromin | NF1 | -4.66771 | 2.53E-05 |
| Alpha-actinin-3 | ACTN3 | -1.942 | 1.48E-05 |
| Dedicator of cytokinesis protein 2 | DOCK2 | -4.68863 | 9.23E-06 |
| Choline transporter-like protein 1 | SLC44A1 | -1.05627 | 0.000259 |
| Methylenetetrahydrofolate reductase | MTHFR | -3.30586 | 0.051116 |
| Membrane-associated phosphatidylinositol transfer protein 2 | PITPNM2 | -8.58612 | 0.000208 |
| Splicing factor_ arginine/serine-rich 19 | SCAF1 | -2.98593 | 0.002543 |
| Dedicator of cytokinesis protein 10 | DOCK10 | -2.96087 | 0.062113 |
| Thrombospondin type-1 domain-containing protein 7B | THSD7B | -3.34977 | 0.001198 |
| Nuclear factor 1 A-type | NFIA | -9.3859 | 6.66E-06 |
| Platelet glycoprotein Ib beta chain | GP1BB | -2.96207 | 0.004638 |
| Histone H2A type 1-A | HIST1H2AA | -2.51795 | 0.000776 |
| Guanine nucleotide-binding protein G(q) subunit alpha | GNAQ | -1.67953 | 0.004988 |
| Creatine kinase B-type | CKB | -5.33995 | 0.088955 |
| Centrosomal protein of 152 kDa | CEP152 | -8.92998 | 0.000155 |
| Spindlin interactor and repressor of chromatin-binding protein | C11orf84 | -8.00452 | 3.94E-06 |
| General vesicular transport factor p115 | USO1 | -1.06531 | 0.02802 |
| Phosphatidylinositol 4-kinase alpha | PI4KA | -1.74599 | 0.095309 |
| HCG1995540_ isoform CRA_b | RAB4B | -3.07752 | 3.12E-05 |
| Nicotinamide N-methyltransferase | NNMT | -4.43479 | 0.087043 |
| Keratin_ type II cuticular Hb6 | KRT86 | -3.23188 | 0.004474 |
| 60 kDa heat shock protein_ mitochondrial | HSPD1 | -3.41188 | 0.030222 |
| Nucleophosmin | NPM1 | -1.59149 | 0.026481 |
| Ras-related protein Rab-8A | RAB8A | -3.14814 | 0.019636 |
| Rab GTPase-binding effector protein 1 | RABEP1 | -1.47151 | 0.00506 |
| FERM_ RhoGEF and pleckstrin domain-containing protein 1 | FARP1 | -1.92728 | 0.037429 |
| Anoctamin | ANO3 | -5.37346 | 0.000408 |
| 3-ketodihydrosphingosine reductase | KDSR | -6.30767 | 0.024137 |
| Centrosomal protein of 135 kDa | CEP135 | -10.778 | 1.04E-06 |
| Fascin | FSCN1 | -2.22732 | 0.047632 |
| 14-3-3 protein zeta/delta | YWHAZ | -2.87886 | 0.000181 |
| Cilia- and flagella-associated protein 61 | CFAP61 | -3.34177 | 0.000234 |
